# Successful Use of Dupilumab in the Treatment of Acquired Perforating Dermatosis Associated with Atopic Dermatitis

**DOI:** 10.1155/2024/6265608

**Published:** 2024-06-19

**Authors:** Niccolò Gori, Eleonora De Luca, Andrea Chiricozzi, Stefania Sfregola, Alessandro Di Stefani, Ketty Peris

**Affiliations:** ^1^Dermatologia, Dipartimento Universitario di Medicina e Chirurgia Traslazionale, Università Cattolica del Sacro Cuore, Rome, Italy; ^2^U.O.C. Dermatologia, Dipartimento di Scienze Mediche e Chirurgiche, Fondazione Policlinico Universitario A. Gemelli IRCCS, Rome, Italy; ^3^Sezione di Anatomia Patologica, Dipartimento di Scienze della Vita e Sanità Pubblica, Università Cattolica del Sacro Cuore, Rome, Italy; ^4^Unità di Anatomia Patologica, Dipartimento di Scienze della Salute della Donna, de Bambino e di Sanità Pubblica, Fondazione Policlinico Universitario A. Gemelli IRCCS, Rome, Italy

## Abstract

Acquired reactive perforating collagenosis is a rare cutaneous disorder characterised by the extrusion of abnormal connective tissue trough epidermidis and/or follicular units. Reactive perforating collagenosis is often associated with systemic diseases in which pruritus is a common symptom (e.g., diabetes and chronic kidney disease). Less commonly, it has been associated with chronic inflammatory dermatoses, including atopic dermatitis, as in this case. In this report, we describe the exceptional case of a 35-year-old man affected by acquired reactive perforating collagenosis associated with atopic dermatitis who was resistant to conventional topical and systemic treatment and experienced complete resolution of clinical signs and symptoms after 12 weeks of treatment with dupilumab. In our patient, the severe pruritus induced by atopic dermatitis likely contributed to the development of acquired perforating collagenosis lesions, which are thought to be a reactive response to chronic scratching and repetitive injury to the skin. Chronic pruritus in atopic dermatitis is known to be driven by type 2 cytokines, including IL-4 and IL-13, and dupilumab, a monoclonal antibody inhibiting IL-4 and IL-13 signalling, has been shown to be effective in the treatment of moderate to severe atopic dermatitis as well as other type 2-driven pruritic dermatological conditions. This case supports the potential use of dupilumab for the treatment of reactive perforating dermatosis.

## 1. Introduction

Acquired reactive perforating collagenosis (ARPC) is classified as a perforating dermatosis, a group of skin disorders characterised by trans epidermal elimination of abnormal connective material (collagen and elastin). It usually occurs in adults, and it is mainly associated with systemic diseases sharing itch as a common symptom, including diabetes mellitus and renal/liver failures [1, 2]. Although less frequently, ARPC has been also associated with itchy inflammatory skin diseases, such as eczema and psoriasis [1].

Itch-scratch cycle is recognised as a fundamental factor in inducing ARPD lesions, as shown by the frequently reported Koebner phenomenon [1]. Consistently, the use of therapies aimed at reducing itching is the mainstay in the treatment of this condition [1, 2].

In this case report, we describe the successful use of dupilumab, a monoclonal antibody interfering with IL-4 and IL-13 signalling, in the treatment of a patient affected by a severe form of ARPD associated to atopic dermatitis, thus expanding the knowledge regarding potential uses of the drug.

## 2. Case Presentation

**Figure 1:**
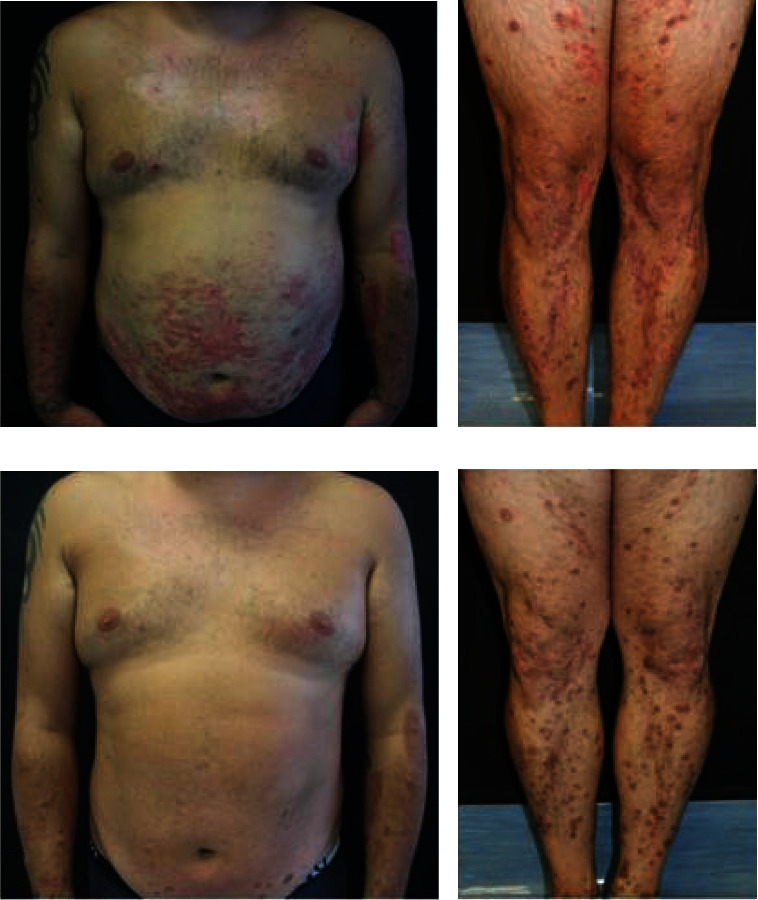
Clinical images. (a, b) At the baseline, the patient presented numerous erythematous/desquamative patches and excoriations intermingled with numerous keratotic plugged papules and nodules that were symmetrically distributed over the trunk and at the limbs. (c, d) An almost complete clearance of both eczematous and papule/nodular lesions, with predominant persistence of postinflammatory, was obtained after 12 weeks of treatment with dupilumab.

A 35-year-old male was examined for a twelve-month history of multiple pruriginous, chronic-relapsing, skin lesions involving the trunk and limbs. Previous treatments with topical and systemic corticosteroids provided only a mild clinical benefit. Patient's medical history included an arteriovenous malformation of the right temporal region involving the meninges and glaucoma. In addition, the patient reported having suffered from food allergies during childhood. Physical examination showed numerous erythematous-scaling patches with eczematous features and excoriations, intermingled with multiple papules and nodules characterised by a central keratotic plug, that were symmetrically distributed on the trunk and lower extremities (Figures 1(a) and 1(b)).

Blood tests including eosinophil count and total IgE were within normal limits and patch tests SIDAPA series resulted negative [3].

**Figure 2:**
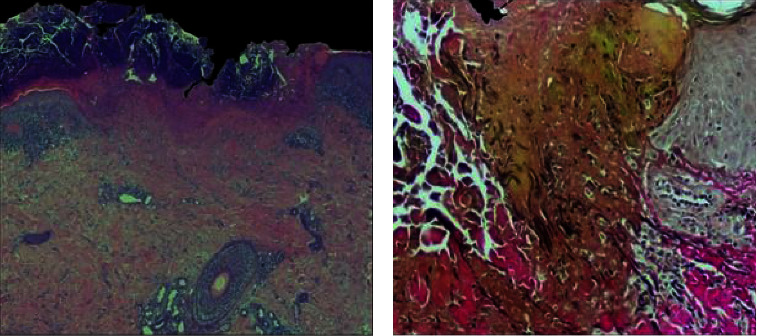
Histopathological images. (a) A broad epidermal ulceration with extensive scale crust. (b) Vertically oriented degenerated and eosinophilic collagen fibers together with black elastic fibers are extruded through the epidermal breach. ((a) Hematoxylin-eosin stain, original magnification: 20x; (b) Verhoef–Van Gieson stain, original magnification: 100x).

Histopathologic examination of a skin biopsy from one erythematous-desquamative patch on the abdomen showed features consistent with chronic eczema, while the histological specimen of one nodule from the right leg revealed a well-delimited area of epidermal necrosis with an overlying thick crust containing cellular debris, keratin, and both extruded collagen and elastic fibers as confirmed by elastic-Van Gieson staining (Figure 2).

The diagnosis of acquired reactive perforating collagenosis (ARPC) associated to severe atopic dermatitis (AD) was made. A systemic treatment with cyclosporine (250 mg/die) allowed to achieve improvement of signs and symptoms after one month. Despite the satisfactory clinical response, cyclosporine was withdrawn because of the occurrence of blood test alterations (dyslipidaemia and liver enzymes alteration) and hypertension. Therefore, dupilumab at the AD-approved dose was initiated. A marked relief from itching (0–10 itch numeric rating scale value from 9 to 2) was reported by the patient after one month of treatment, while a marked clearance of both eczematous and papulonodular lesions was achieved after 12 weeks, with persistence of postinflammatory hyperpigmented macules and rare erythematous macules and patches mainly on the lower legs (Figures 1(c) and 1(d)).

## 3. Discussion

Management of perforating dermatosis is essentially based on mitigation of pruritus and treatment of any associated comorbidities. Topical steroids, antihistamines, and moisturisers are commonly used in mild ARPC, while in severe forms of disease, various systemic agents, including steroids, retinoids, tetracyclines, phototherapy, dapsone, methotrexate, and allopurinol, may be used [1, 2].

In our patient, the severe itch related to AD likely contributed to the development of ARPC lesions, which are supposed to represent a reactive response to chronic rubbing and repetitive trauma to the skin [1].

Chronic pruritus in AD is mainly mediated by the Th2-signature cytokines, interleukin (IL)-4, and IL-13, which act directly on itch sensory neurons to promote itching. In addition, IL-4 enhances neural hypersensitivity to various other molecules such as histamine, chloroquine, IL-31, and cytokine-thymic stromal lymphopoietin, known to be strong pruritogens [4].

Dupilumab, a subcutaneous monoclonal antibody inhibiting the signalling of IL-4 and IL-13, has been the first biological agent being approved for AD, but clinical trial and real-life experiences revealed the efficacy of dupilumab also for the treatment of other pruritic skin disorders, including prurigo nodularis, bullous pemphigoid, lichen planus, and pruritus sine materia [5].

Notably, a few recently published real-world experiences reported dupilumab effectiveness in the treatment of individuals with ARPC, associated or not with AD [6, 7].

Considering the safety profile of dupilumab also demonstrated in frail patients affected by multiple comorbidities, including diabetic and kidney failure, it could be a valuable potential treatment option for severe ARPC-associated itching.

## Data Availability

All data generated or analyzed during this study are included in this article. Further inquiries can be directed to the corresponding author.
